# Supplementary material for: A physics-informed deep learning liquid crystal camera with data-driven diffractive guidance
Source: Commun Eng. 2024 Mar 13;3:46. doi: 10.1038/s44172-024-00191-7 (PMC10956035; doi:10.1038/s44172-024-00191-7)
Supplement: Supplementary file 2 — Supplementary Information [file 44172_2024_191_MOESM2_ESM.pdf]

Supplementary information: A Physics-Informed Deep Learning Liquid-Crystal Camera with Data-Driven Diffractive Guidance

JIASHUO SHI[1,2], TAIGE LIU[1,2], LIANG ZHOU[1,2], PEI YAN[1,2,3], ZHE WANG[1,2], XINYU ZHANG[1,2,*]

AFFILIATIONS

[1]National Key Laboratory of Science and Technology on Multi-spectral Information Processing, Huazhong University of Science and Technology, Wuhan 430074, China

[2]School of Artificial Intelligence and Automation, Huazhong University of Science and Technology, Wuhan 430074, China

[3]School of Computer Science and Engineering, Nanyang Technological University, Singapore 628798, Singapore

**Corresponding author: x_yzhang@hust.edu.cn*

This supplemental document is organized as follows:

In Supplementary Note 1, we expand the experimental validation section to include a more diverse set of scenarios. This will provide a more comprehensive assessment of the method's robustness and effectiveness across a range of applications.

In Supplementary Note 2, we show how much prediction accuracy and time the proposed all-optical configuration saves in comparison to conventional digital post-processing approaches.

In Supplementary Note 3, we present a comprehensive illustration of the mode switching characteristics within the liquid crystal (LC) layer of the 1mm aperture-patterned electrode configuration.

In Supplementary Note 4, we include a schematic diagram of the aluminum electrode, along with its dimensions, to enhance the visual representation and comprehensibility of this element in the experimental setup.

Supplementary Note 1:

Supplementary Figure 1 presents results from experiments conducted in various scenes to confirm the effectiveness of the proposed model. The figure showcases different scenes (subfigures 1-8) with corresponding diffractive network predictions of the light field. In subfigure 1, the scene features no target of interest (ToI), while subfigures 2 to 3 include the ToI located within the [R_4, R_5] region, and subfigures 4 to 8 have the ToI situated in the [R_6, R_7] region. To test the model's robustness, yellow objects resembling the features of the ToI were introduced at different positions within the scenes. Additionally, the ToI is laterally displaced within the scenes, and even when it reached the edge of the scene, the proposed model demonstrated a relatively accurate prediction, highlighting its robust performance.

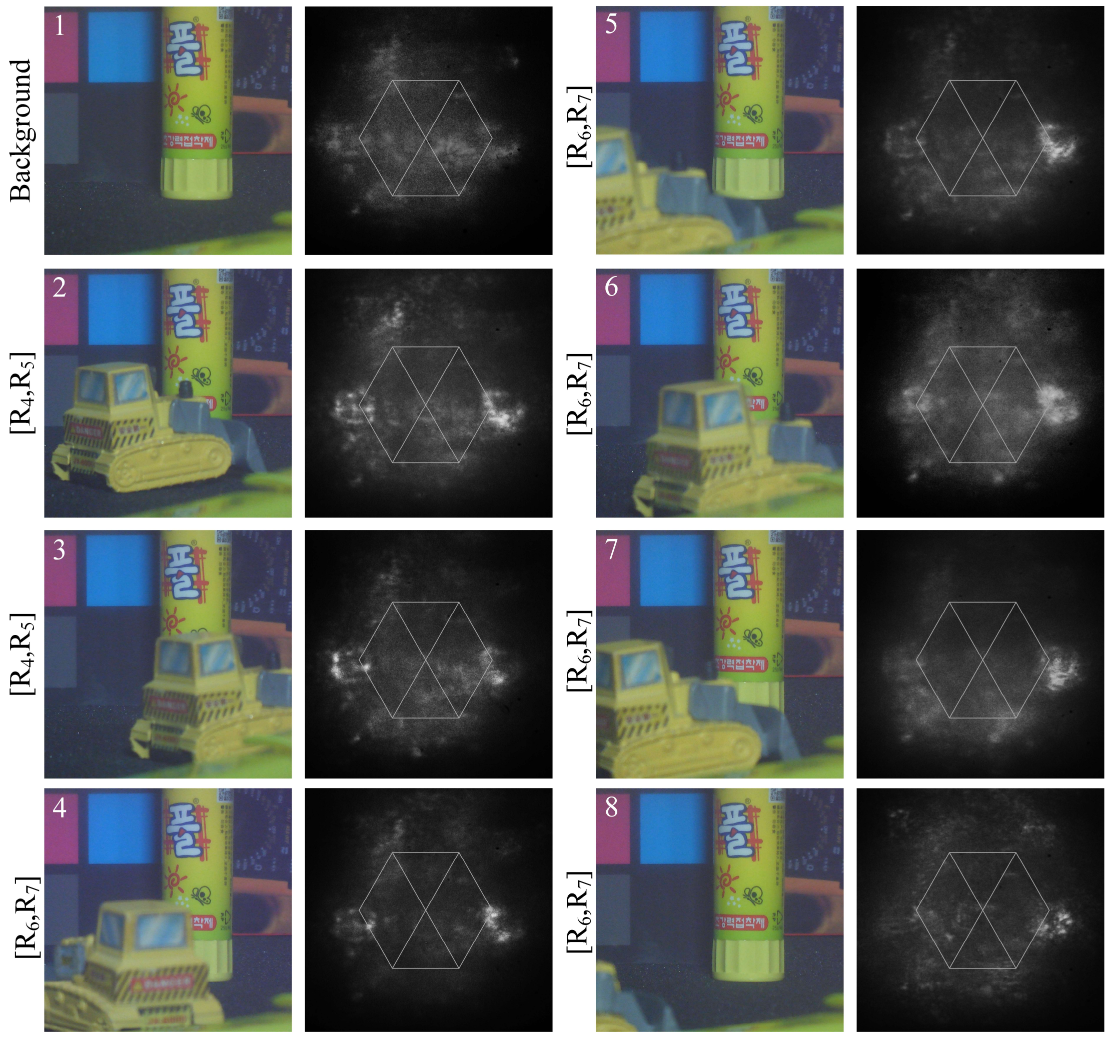

Supplementary Figure 1: Experimental confirmation of learning-based diffractive guidance in other scenes.

Supplementary Note 2:

In Supplementary Figure 2, we provide a comparative analysis of the prediction time and accuracy for three different methods: the full connection neural network (FCNN), convolutional neural network (CNN), and the proposed method, with each network comprising three layers. Each training curve represents the results obtained after six rounds of training, with individual Epoch tracked for prediction accuracy variance, and average values. Notably, when the proposed optical structure is implemented, it demonstrates a significant advantage in prediction time, which is unmatched by the traditional methods.

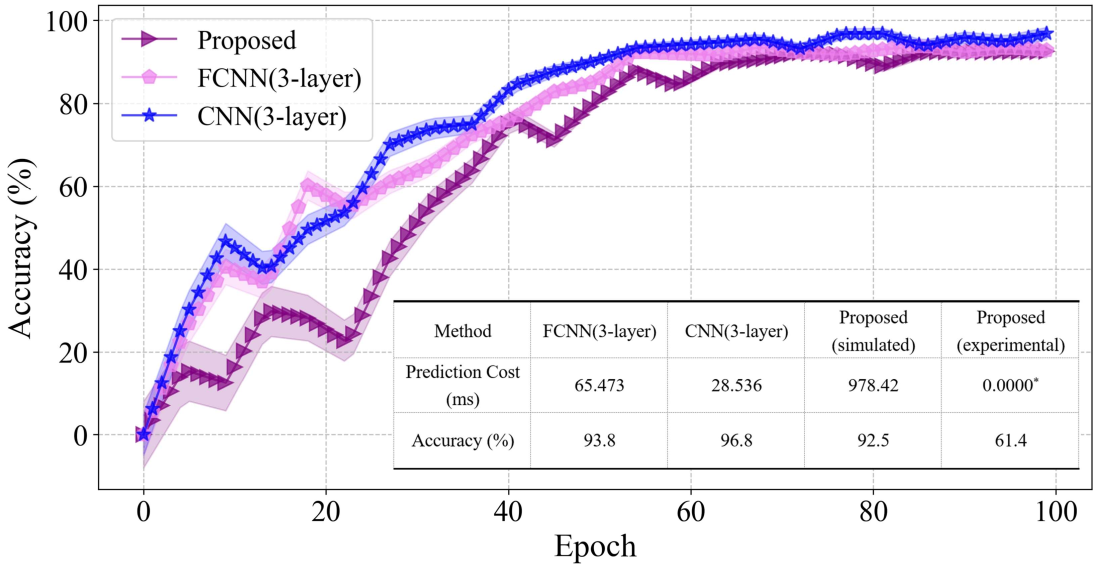

Method	FCNN(3-layer)	CNN(3-layer)	Proposed (simulated)	Proposed (experimental)
Prediction Cost (ms)	65.473	28.536	978.42	0.0000*
Accuracy (%)	93.8	96.8	92.5	61.4

Supplementary Figure 2: Performance comparison of three methods.

"" means that it can be ignored in the current measuring range.*

Supplementary Note 3:

Supplementary Figure 3 presents a comprehensive illustration of the mode switching characteristics within the liquid crystal (LC) layer of the 1mm aperture-patterned electrode configuration. The simulation captures the modal responses of the LC layer under varying voltage conditions, namely 5V, 20V, and 30V. The corresponding electric potential distributions are visualized, providing insight into how the electric field is modulated within the LC layer. Additionally, the figure showcases the distribution of LC molecule orientation vectors, demonstrating the dynamic response of the LC layer to different voltage conditions.

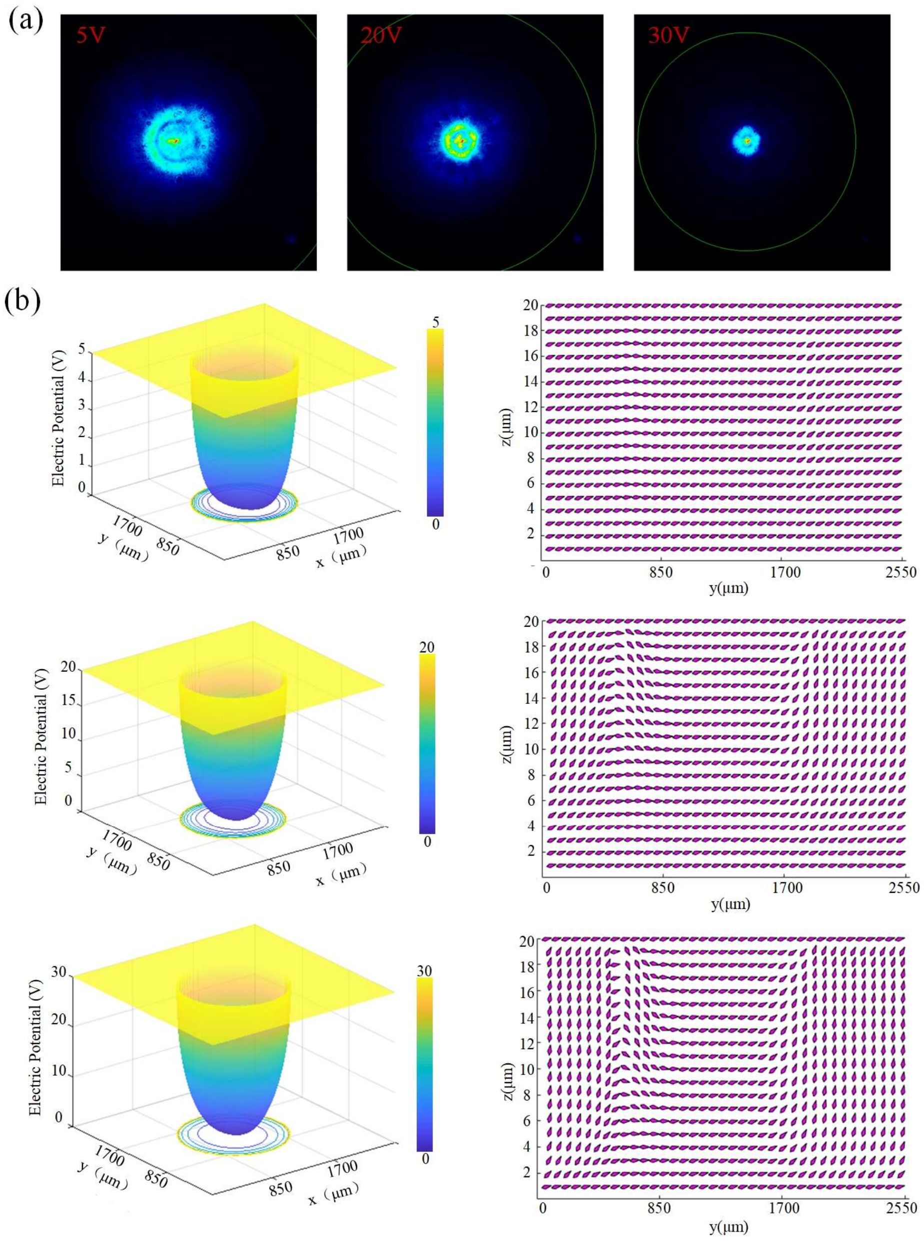

Supplementary Figure 3: Mode switching of the electric potential distributions across the LC layer.

Supplementary Note 4:

We include a schematic diagram of the aluminum electrode, along with its dimensions, to enhance the visual representation and comprehensibility of this element in the experimental setup.

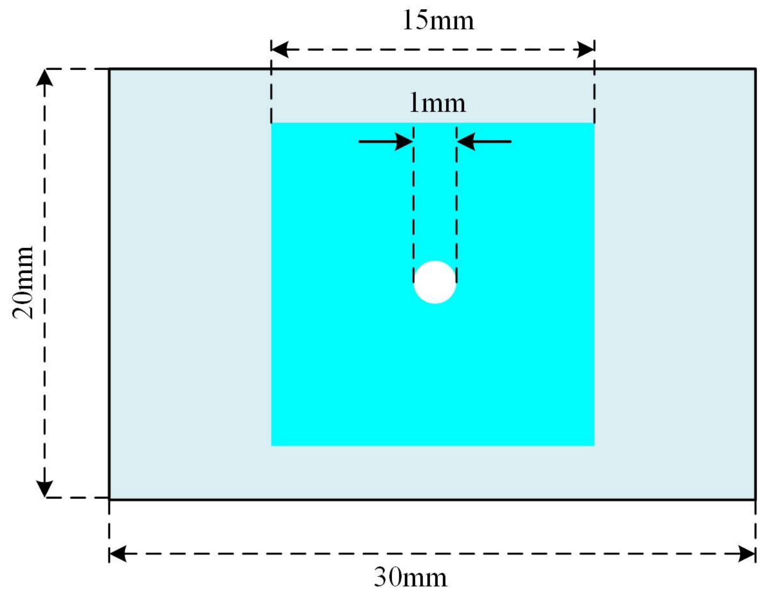

Supplementary Figure 4: Schematic of circular-patterned aluminum electrode.
