# Supplementary material for: A physics-informed deep learning liquid crystal camera with data-driven diffractive guidance
Source: Commun Eng. 2024 Mar 13;3:46. doi: 10.1038/s44172-024-00191-7 (PMC10956035; doi:10.1038/s44172-024-00191-7)
Supplement: Supplementary file 3 — Description of Additional Supplementary Files [file 44172_2024_191_MOESM3_ESM.pdf]

Description of Additional Supplementary Files

File name: Supplementary Movie 1

Description: Video shows results from experiments conducted in various scenes to confirm the effectiveness of the proposed model. The video showcases different scenes with corresponding diffractive network predictions of the light field.
